# Emotional Well-Being and Cognitive Function Have Robust Relationship With Physical Frailty in Institutionalized Older Women

**DOI:** 10.3389/fpsyg.2020.01568

**Published:** 2020-07-16

**Authors:** Guilherme Eustáquio Furtado, Adriana Caldo, Ana Vieira-Pedrosa, Rubens Vinícius Letieri, Eef Hogervorst, Ana Maria Teixeira, José Pedro Ferreira

**Affiliations:** ^1^University of Coimbra – Research Unit for Sport and Physical Activity (CIDAF, UID/PTD/04213/2019), Faculty of Sport Science and Physical Education (FCDEF-UC), Coimbra, Portugal; ^2^Multidisciplinary Research Nucleus in Physical Education (NIMEF), Physical Education Department, Federal University of Tocantins (UFT), Tocantinópolis, Brazil; ^3^Applied Cognitive Research NCSEM, Loughborough University, Loughborough, United Kingdom

**Keywords:** frail older adults, subjective well-being, mental health, mild cognitive impairment, psychological frailty, depression, happiness

## Abstract

**Introduction:**

Frailty associated to core dimensions of psychological well-being (PwB) has appeared as a possible new frailty phenotype named psychological frailty, implying a parallel to physical frailty (PF). Very little is known about the associations between mental well-being, especially emotional, mood, and self-perception dimensions, and the frailty syndrome in institutionalized older populations. The present study aims to examine the interlink between the PF phenotype and the core dimensions of PwB in Portuguese institution-dwelling older women.

**Methods:**

Cross-sectional data were collected. A total of 358 older women, aged 75 years or more, were recruited from four nursing homes within the city of Coimbra and asked to complete a sociodemographic and a general health assessment survey. The main PwB dimensions were assessed in all participants: (i) global cognitive status was assessed using The Montreal Cognitive Assessment (MoCA) Neuropsychology Test, (ii) self-perception was screened using the General Self-Efficacy Scale (GSES) and Global Self-Esteem Scale, (iii) CES-D of depression and Perceived Stress Scale (PSS) were used to screen mood states, and (iv) subjective happiness, satisfaction with life, and attitudes to aging psychometric rating scales were used to screen for emotional well-being. The syndrome of PF was assessed using Fried’s PF phenotype that includes weight loss, weakness, slowness, exhaustion, and low physical activity (PA) level assessments.

**Results:**

Frail older women had a poor score in all PwB outcomes, except for global self-esteem and satisfaction with life. A hierarchical regression model analysis showed that global cognitive status and emotional well-being of subjective happiness and attitude to aging showed a significant negative relationship with PF in both unadjusted and adjusted models (explaining 34 and 40% of variance, respectively).

**Conclusion:**

Emotional well-being and global cognitive performance are strongly associated with PF. Implementing active lifestyle interventions to improve positive psychological outcomes using geriatric assessments could assist in the older institutionalized patients’ physical and mental health care.

## Introduction

Frail populations are at an increased risk for adverse negative health consequences (Middleton et al., 2008). Disability, morbidity, institutionalization, and hospitalization are likely outcomes of this clinical time-progressive form of unsuccessful aging (Clegg et al., 2013). In the frail person, the order of adverse events occurs earlier and faster, significantly affecting their psychological adjustment and quality of life (Gobbens et al., 2013; Kanwar et al., 2013). A contemporary approach to the concept of physical frailty (PF) made by specialists recognizes it as a syndrome associated with aging that causes increased vulnerability to stressors due to deficiencies between various interrelated physiological systems, leading to a decline in homeostasis (Morley et al., 2013). The concept of PF established by Fried et al. (2001) is understood as a robust construct and has five components that comprised perceived exhaustion, weight loss, and also low levels of hand grip strength, gait speed, and physical activity (PA). It is assumed to be very helpful for health professionals and researchers and to comprehend the heterogeneity of health trajectories linked to frailty (Morley et al., 2013).

Despite the considerable evolution of the concept of frailty carried out to date, which culminated in the development of other models and frailty indexes, recent observational studies have presented other health-related dimensions that can better explain this syndrome (Kelaiditi et al., 2013). The recently coined concept of cognitive frailty (CF) has been described to mediate the probability of numerous types of neuropsychological impairments (Buchman and Bennett, 2013). The concept of CF proposes a communality to frailty syndrome, with loss of adaptability in the domain of cognitive skills and decreasing resilience to internal and external factors, and also denotes a linkage to PF (Canevelli et al., 2015). Several research on expanded PF have concerned cognition domain as a prospective factor strongly affecting health-related geriatric outcomes (Panza et al., 2015).

As with the concept of CF, the term “psychological frailty” appears as a new frailty sub-phenotype and implies a parallel to PF but in the brain activities dimension and suggesting a relationship between the two (Fitten, 2015). Recently, the conceptual origins of psychological well-being (PwB) dimensions were revisited, which gave rise to an update of the concept focusing on six emerging areas of contemporary psychology: (i) perception of well-being changes throughout human development, (ii) personality correlates of well-being, (iii) well-being linked to experiences in family life, (iv) well-being associated to employment activities and other work occupations, (v) interconnections between physical and mental health, including biological aspects, and (vi) clinical and intervention research involving well-being in different segments of society (Ryff, 2014).

The phenotype of PF has been characterized by factors linked with several negative psychosocial facets that manifest throughout the aging progression and have already been studied, but the difference is that the PF condition seems to worsen some psychological aspects (Freitag and Schmidt, 2016). In addition to cognitive status, studies aimed to analyze the multifaceted interactions between different PwB markers and frailty revealed that motivation (i.e., self-efficacy and attitudes), negative and positive feelings of mood (i.e., depression and stress), and emotional well-being were identified as the core dimensions of PwB t in older populations (McAuley et al., 2006; Dent and Hoogendijk, 2014; Gale et al., 2014; Freitag and Schmidt, 2016).

Each individual has a single genotype and a set of lifespan involvements that will fare in terms of general health and chance of disease (McEwen, 2015). Thus, distinct mood states are the most important contributors to PwB and reflect in the self-perception of physical health (Thoits, 2011). Psychosocial stress, for example, is associated with the onset and the progression of many and costly comorbidities, including chronic pain conditions linked to functional disabilities (Muscatell et al., 2015). Positive self-esteem, on the other hand, is seen as a protective factor that contributes to a highly positive physical self-perception in frail subjects (McAuley et al., 2005). High perceptions of self-efficacy appear to be associated with good levels of motor skills in frail people (Chou et al., 2012). Adverse negative conditions of physical health can influence older people’s subjective perception of positive feelings, mostly when they determine a reduction in their levels of subjective well-being and their individual perception of their general health (Cho et al., 2011; Wu et al., 2013).

In spite of the critical contribution of core PwB s to explain PF, not many studies used different measurements to describe how frail populations evaluate their levels of subjective well-being, self-perceptions, mood states, and how these are related to PF. A previous research looking at these associations was done in community and in hospital-based populations (Dent and Hoogendijk, 2014). Other recent findings show that some domains of subjective well-being perception decreased by a PF identity crisis may mediate a self-reported health status in older populations (Andrew et al., 2012). However, very little is known about this relationship in specific populations, e.g., those living in nursing homes. Another important question is the fact that most studies used the Mini-Mental State Exam to assess the general cognitive profile (Furtado et al., 2018). However, in this research, we opted for the Montreal Cognitive Assessment (MoCA) due to its efficiency in screening older individuals with mild cognitive impairment and the careful manner in which the validation for the Portuguese population has been carried out (Duro et al., 2010; Freitas et al., 2014).

The context of nursing homes provides a crucial location for the study of these connections due to the patients’ heterogenic condition in terms of physical status, comorbidities, and psychological outcomes. Thus, this study aimed to analyze the association of PF with core PwB dimensions, specifically, to explore the relationship between PF and subjective and mental well-being in institutionalized older women.

## Materials and Methods

Data collection of all outcome measures was organized by the principal investigator and performed by independent specialists who had been extensively trained by the research team. The same evaluators for each study domain performed the data collection in all study participants using a face-to-face approach. Individual attention was provided to the participants with interpretation queries, and questions were read from a standard sheet to avoid response bias.

### Initial Procedures and Study Design

A cross-sectional design using a survey on frailty incidence in institution-dwelling older individuals living in the region of Coimbra City (Portugal) was followed. The participants consisted of a subgroup within a previously published study (Furtado et al., 2019). A total of 10 centers for social and health care (CSH) were selected as eligible to participate in the first study phase. After the visits to the homes to communicate the purpose of the study and to verify the eligibility selection criteria, five CSH were selected to participate in this study.

### Eligibility Criteria

All eligible participants that took part in the study voluntarily signed a written informed consent form. In the first phase, all female participants aged 75 years and above agreed to take part on this research and, with their prescribed medications controlled, were admitted to the study. The specific exclusion criteria were: (i) the existence of some type of illness disorder that could hinder the assessment of autonomy, such as musculoskeletal impairment (i.e., advanced attrite or arthrosis), cardiomyopathy, cardiorespiratory illness, and other clinical settings that might prevent functioning (i.e., recent fractures); (ii) mental disorders (i.e., psychosis, depression, anxiety, and dementia), low visual acuity and hearing ability, and classified as morbidly obese; (iii) identification of any drug therapy in the process of adaptation or deregulation that may cause deficit of attention and/or substantial changes in motor activities (i.e., anti-depressants, betablockers, and anxiolytics).

### Sample Selection Statement

The first eligible participants included in the study were 486 (100%) institution-dwelling old adults aged over 75 years. After applying the sample selection criteria, 128 participants (25%) were excluded or dropped out due to (i) physical impairment associated with musculoskeletal disorders and joint or muscle pain in the performance of specific movements or tests (*n* = 44), (ii) closed diagnosis of early stage dementia or other mental disorders (*n* = 29), (iii) severe uncorrected impairment of hearing or visual functions that made it impossible to perform all tests (*n* = 17), (iv) need of palliative health care or special nutritional support, with medical indications not to participate in the study (*n* = 19); (v) participants who dropped out when applying the tests (*n* = 20); and (vi) inconsistent data (*n* = 08). The final number of participants was 358.

### Ethical Report

This study respected the Health Sciences Portuguese Resolution (Article 4th; Law number 12/2005, 1st series) on ethics in research and complied with the guidelines for research with human beings in the Helsinki Declaration (Petrini, 2014). First, the study protocol was approved by the Multidisciplinary Ethical Committee of the Faculty of Sport Science and Physical Education (reference code number CE/FCDEF-UC/000202013). All CSH directors and participants signed the informed consent form in the first study approach. This document explains in detail the phases of the study, how to collect, treat, and analyze the obtained data, and the criteria used for identity privacy.

## Outcome Measures

The PF phenotype and its five components were the primary outcomes and the domains of PwB were the secondary outcomes. Sociodemographic, general health status, and anthropometric measures that showed significant statistical differences in comparison to PF subgroups were understood as possible confounders and were entered as covariates in a subsequent analysis.

### Physical Frailty Screen

A negative evaluation in one or two criteria classified the participants as pre-frail, in three or more as frail, and as non-frail when the subject had a void in any of the five criteria, forming a dichotomic categorical classification. Fried’s PF protocol was used (Fried et al., 2001):

(i) Weight loss: the medical record was consulted to check if the participant had unintentionally lost 4 kg of weight or more in the last 6 months.
(ii) Exhaustion: consisted in a self-report measure that was evaluated through the agreement of questions 7 (*I felt that everything I did was an effort)* and 20 (*I could not get going*) of the CES-D questionnaire (Gonçalves et al., 2014).
(iii) Muscle weakness: analyzed using the handgrip strength test. This test uses a portable dynamometer device (Lafayette, model 78010, United States). The participant grabs the device in one hand, with the arms extended next to the trunk. At the signal, the participant squeezes the device with maximum effort, using an isometric contraction force, for 5 s to acquire stability in the measure. The best result of two attempts was taken as an official measure for scoring purposes (Syddall et al., 2003). The participants who did not achieve proficiency in the test and those who classified themselves below 20% [adjustment by body mass index (BMI) and sex] were categorized as individuals who have muscle weakness (Fried et al., 2001).
(iv) Slowness of gait: measured by using the “4.6 m test” where the participant had to walk the distance of 4.6 m, without assistance, in a straight line. The time in seconds characterizes the registration measure for this test, adjusted by the participants’ gender and height. The participants had two attempts to perform the test, and the cutoff values used were the ones suggested by the original PF protocol (Fried et al., 2001).
(v) Low levels of PA were assessed using the International PA Short Form assessment (IPAQ-sf). The participants were categorized as “inactive” and “minimally” active according to the IPAQ-sf criteria if they had a positive score for the PF status.

In addition, the prevalence of each of the five components was calculated to generate the continuous variable of PF composed score with a range from 0 to 5 points, where the higher values represent a higher frailty status.

### Screen of PwB Dimensions

The psychometric tests described below were chosen because they had been validated in the Portuguese population and characterize the core PwB dimensions described in the concept of psychology of frailty as previously defined (Fitten, 2015):

(i) Cognitive status: The Montreal Cognitive Assessment was used to evaluate global cognitive performance. The MoCA assesses different areas of cognitive function: language, working memory and task concentration, spatial orientation, executive functions, and visuospatial abilities. The maximum score to be achieved in the MoCA is 30 points, and according to the validation values presented for the Portuguese population, if the participant obtains a score below 22 points, he/she can be screened as having mild cognitive impairment or dementia (Freitas et al., 2013).
(ii) Mood states: CES-D scale was used to assess the depression state. Each one of the 20 questions has four answer options in a Likert-type scale, with global scores between 0 and 60 points. The highest scores correlate with more depressive sign in the last week (Gonçalves et al., 2014). The Perceived Stress Scale (PSS) assesses the perception of stressful experiences. This scale has 14 items; seven have a positive connotation and the other seven a negative direction. The scores can vary between 14 and 70 points, and the higher scores attained by the participants reveal greater symptoms of stress (Taylor, 2015).
(iii) Self-perception: The Rosenberg Self-Esteem Scale (RSES) analyzes the evaluative dimension of self-concept. The RSES has 10 questions with a Likert-type scale and four answer options, with global scores between 10 and 40 points. The higher scores reveal greater self-esteem levels (McKay et al., 2014). The General Self-Efficacy Scale (GSES) was used to evaluate resilience and optimism to deal with situations and the ability to solve everyday life problems effectively. When answering the questions, the participants can achieve a score ranging from 10 to 40 points. The higher scores reveal greater GSES levels (McAuley et al., 2005).
(iv) Emotional well-being: The Satisfaction With Life Scale (SWLS) assesses general and personal judgments of satisfaction with one’s own life. The five-item scale results are scored between one and 35 points. A high score achieved by the respondents represents greater personal satisfaction with one’s own life at the present moment (McKay et al., 2014). The Happiness Face Scale (HFS) consisted of a graphical scheme where for each face one letter is assigned, in which letter A (seven points) is considered as the maximum and letter G as the minimum (one point). The participant has to identify with one of the faces, depending on his/her state of happiness (Andrews and Withey, 1976). The Attitudes to Aging Questionnaire (AAQ) assesses specific feelings toward the aging process as an intrapersonal experience from the older point of view, taking into account their expectations, worries, emotions, and behavior. The AAQ contains 24 items and total scores range from 8 to 40 points. The higher scores express a more positive attitude toward our own aging process across the life (Low et al., 2013).

### Anthropometric and Sociodemographic Measures

Chronological age was treated as a continuous variable. Marital state was assessed as a four-category variable: single, married, widowed, and divorced. The level of education was collected for each participant, classified in number of years, and analyzed as a continuous variable. Standardized and validated techniques were respected, and anthropometric data collection procedures were as previously described (Chumlea and Baumgartner, 1989) and included the following measures: (i) weight or body mass was measured by a portable scale with a precision of 0.1 kg and (ii) stature was determined using a portable stadiometer with a precision of 0.1 cm (Seca Portable Anthropometric Body meter^®^ model 208, Germany). BMI was calculated according to the formula BMI = weight/height^2^.

### General Health Profile

Levels of comorbidity were assessed by the Charlson Comorbidity Index (CCI). The CCI evaluated the weight of several diseases. Each disease has a specific score, varying from 1 to 3 points, and the sum of the total values related to the diseases recorded in the participants’ medical record forms a single score, treated as a continuous variable. One point for each additional 10 years is added to the initial score that has been shown to predict 1- and 10-year mortality. A recent study carried out a successful update of the index to 12 comorbidities (Quan et al., 2011). The use of chronic or acute medication of each participant was systematically checked with the medical staff, and polypharmacy was considered according to the Classification System of Human Medicine in Portugal when the participant uses more than three drugs in a chronic treatment.

### Statistical Analysis

The initial assumptions of data were verified by visual inspection of the normality plots and the Shapiro–Wilk statistical test. Continuous variables were reported by their medians and 25th and 75th percentiles, whereas categorical variables were reported by relative and absolute frequencies. A comparison of quantitative variables between the frail subgroups was performed using ANOVA or Kruskal–Wallis, depending on whether the variables were found to be normally distributed, which was ascertained by employing the Shapiro–Wilk tests. Bonferroni correction test was performed to adjust the comparisons analysis. In this study, the PF composed score was assumed as a dependent variable, following previous publications (Ávila-Funes et al., 2011). The association between the groups of qualitative variables was assessed using chi-square tests. Partial correlations between the PF and the PwB were computed together with partial correlations controlling for the assumed covariates (cognitive status, comorbidities, marital status, and height). The PwB variables that showed stability in significance after controlling for covariates in the partial correlation model were taken from the regression analysis, respecting the statistical assumption (Jeong and Jung, 2016). The relationships between PF and PwB were analyzed using a hierarchical stepwise regression model. In this model of analysis, the PwB outcomes were assumed as independent variables. A total of three independent linear regressions were selected over a hierarchical stepwise and multiple-regression analysis, considering the previous hypothetical and theoretical assumption that CES-D and MoCA showed a strong statistical significance with PF (Jeong and Jung, 2016). In these, cognitive status was introduced as a first block in the model. Secondly, the depression state of CES-D was entered together with cognitive status. Lastly, all other PwB indicators were entered in the statistical models to possibly explain the assumption of regression model maximal variance. The unadjusted bivariate model 1 simply included the dependent variable of PF composed score and the independent variable of PwB outcomes. Model 2 was further adjusted for variables of height, marital status, and comorbidity. The degree of the associations was discussed according to the magnitude of the correlations, which are understood as robust (*r* = 0.7–0.8), strong (*r* = 0.5–0.7), moderate (*r* from 0.3 to 0.4), small (0.1–0.2), and trivial (*r* < 0.1) (Hopkins et al., 2009). The software R 3.3.1 and IBM SPSS 22.0 were used for all statistical treatments. The statistical significance level adopted in this study was *p* < 0.05.

## Results

The descriptive characteristics of the participants for all variables by frail subgroups are presented in Table 1. According to preliminary checks, the variables that did not show normality were marital state, MNA, weight, BMI, and medication use. For those, comparative analysis was performed using Kruskal–Wallis test (*p* < 0.05). For all the others, ANOVA test was used. A total of 78 participants were categorized as non-frail (16%), 136 as pre-frail (38%), and 144 as frail (46%). Sociodemographic data showed that the participants have a median age of 83.0 (76.0–88.0) years, low median (3rd grade) academic achievement levels according Portuguese classification, and also mostly without a husband (94%). There were significant statistical differences between frail subgroups in marital status (*p* = 0.028) and anthropometric measure of height (*p* = 0.008), but not weight. Regarding to general health status, the mean scores of the total sample reflected a high prevalence of comorbidities and mortality with a median of 7 (6–9), with significant statistical differences between frail subgroups (*p* = 0.013). A high incidence of polypharmacy and a clear trend for increased polypharmacy in the frail subgroup were revealed. Taking into account the assumptions initially established for this study, marital state, height, and CCI variables were classified as covariates in the analysis of the correlation models. In addition, a preliminary comparison analysis performed by “nursing homes” subgroups for all variables showed that no significant statistical differences were found, which means that it did not enter as a covariate in the adjustment models (*p* < 0.05).

**TABLE 1:** Characterization of the total sample and comparison by physical frailty subgroups for sociodemographic, anthropometric, and general health status.

**Variable**	**Total sample (*n* = 358, 100%)**	**Non-frail (*n* = 78, 21%)**	**Pre-frail (*n* = 136, 38%)**	**Frail (*n* = 144, 40%)**	***p* value**
**Sociodemographic (M1:3)**					
Chronological age (years)	83.0 (76.0; 88.0)	82.0 (77.0; 88.0)	83.0 (76.0; 89.0)	83.0 (76.0; 87.0)	0.954
Level of education (degree)	3.0 (3.0; 4.0)	4.0 (3.0; 6.0)	3.0 (3.0; 4.0)	3.0 (2.0; 4.0)	0.060
**Marital state (*n*,%)**					
Single	30 (25.4)	6 (31.6)^b,c^	11 (24.4)	13 (24.1)	0.028
Married	7 (5.9)	4 (21.1)^b,c^	1 (2.2)^a^	2 (3.7)	
Widowed or divorced	81 (68.6)	9 (47.4)^b,c^	33 (73.3)^a,c^	39 (72.2)^a,b^	
**Anthropometric (M1:3)**					
Weight (kg)	66.1 (57.2; 71.4)	65.7 (58.6; 77.9)	65.7 (56.8; 71.4)	66.5 (53.1; 70.5)	0.951
Height (m)	1.5 (1.5; 1.6)	1.6 (1.5; 1.6)	1.5 (1.5; 1.6)	1.5 (1.5; 1.5)	0.008
Body mass index (M1:3)	29.0 (24.6; 31.5)	27.0 (24.6; 30.1)	29.2 (24.4; 31.6)	30.2 (25.3; 32.3)	0.207
**General health state**					
Charlson Comorbidity Index (0–10 pts, M1:3)	7.0 (6.0; 9.0)	8.4 (6.0; 10.0)^b^	7.0 (6.0; 8.0)^a,c^	8.3 (7.0; 9.0)^b^	0.013
Mini-Nutritional Assessment (0–10 pts, M1:3)	24 (19, 25)	23 (17, 21)	24 (18, 24)	23 (22, 25)	0.918
**Medication use, per day (*n*,%)**					
I use more than three	108 (91.5)	17 (89.5)	43 (95.6%)	48 (88.9)	0.434
I use three or less	10 (8.5)	2 (10.5)	2 (4.4)	6 (11.1)	

*M1:3, median, first, and third quartile; *n*,%, number and percentage of participants; pts, points. ^*a*^Significant differences compared to non-frail. ^*b*^Significant differences compared to prefrail. ^*c*^Significant differences compared to frail. ANOVA or Kruskal–Wallis test was computed depending on assumption of data.*

Table 2 shows the characteristics of the study sample and the comparison analysis by frail subgroups according to the PwB indicators. According to the initial normality verification, variables such as AAQ, CES-D, and MoCA did not fulfill the normality assumptions, and a comparison analysis was performed using Kruskal–Wallis test (*p* < 0.05). For all the other PWB variables, ANOVA test was used for comparison. The results showed significant statistical differences for cognitive profile of MoCA (*p* < 0.001), mood states of CES-D scale (*p* = 0.001), and stress scale of PSS (*p* = 0.003) as well as lower scores for self-perception of GSES (*p* = 0.017), attitudes to aging as assessed with the AAQ (*p* = 0.005), and subjective well-being of HFS (*p* = 0.037). No significant statistical differences were found for SWLS and RSES. Independent of directions of the scale’s quotation, the statistically significant results indicated worse values for the frail subgroup.

**TABLE 2:** Comparison scores of psychological well-being outcomes by physical frailty subgroups.

**Psychological well-being status**	**Total sample (*n* = 358, 100%)**	**Non-frail (*n* = 78, 21%)**	**Pre-frail (*n* = 136, 38%)**	**Frail (*n* = 144, 40%)**	***P* value**
Montreal Cognitive Assessment (0–30 pts)	17.0 (13; 21)	22.0 (21; 27)^b,c^	19.0 (14; 22)^c^	14.0 (10; 19)^a,b^	<0.001
CES-D Depression Scale (0–60 pts)	22 (16; 28)	17 (12; 27)^c^	18 (14; 24)^c^	24 (20; 30)^b^	0.001
Perceived Stress Scale (0–60 pts)	27 (22; 31)	22 (14; 27)^b,c^	26 (22; 32)^a^	27 (26; 32)^a^	0.003
Global Self-Esteem Scale (10–40 pts)	22 (19; 25)	22 (17; 26)	23 (19; 25)	22 (20; 25)	0.928
General Self-Efficacy Scale (10–40 pts)	30 (25; 34)	33 (29; 36)^b,c^	30 (25; 34)^a^	30 (25; 31)^a^	0.017
Attitudes to Aging Questionnaire (8–40 pts)	73 (64; 88)	81 (73; 102)^b,c^	76 (67; 89)^a,c^	68 (59; 80)^a,b^	0.005
Satisfaction With Life Scale (1–35 pts)	23 (20; 28)	24 (19; 27)	24 (22; 29)	22 (20; 27)	0.171
Subjective Happiness Face Scale (1–7 pts)	3 (2; 5)	4 (3; 7)^c^	4 (3; 5)^c^	3 (2; 4)^a,b^	0.037

*CES-D, Center of Epidemiology Studies for Depression; pts, points. ANOVA or Kruskal–Wallis test was computed depending on assumption of data. ^*a*^Significant differences compared to non-frail. ^*b*^Significant differences compared to prefrail. ^*c*^Significant differences compared to frail.*

Table 3 shows the Spearman’s rank and partial correlations, controlling for potential confounders (marital status, height, and CCI). The data were analyzed by the five nursing homes (categorical variables) that were part of the study and did not present significant differences for all biosocial and general health status variables, so it did not enter as a covariate in the adjustment models (*p* < 0.05). A significant and stable correlation emerged between PF and all PwB indicators, except with SWLS and RSES. After applying a statistical adjustment, the correlations were moderately attenuated or increased, but several important associations persisted. In the correlations between the PwB variables, it was verified that all values were lower than *r* = 0.70, indicating that the assumption of non-multicollinearity among factors (taking into account the introduction of these variables in the regression model) was not violated.

**TABLE 3:** Spearman and equivalent partial correlations between physical frailty composed score and psychological well-being outcomes (*n* = 358).

**Variables**	**1**	**2**	**3**	**4**	**5**	**6**	**7**	**8**
1. Physical Frailty Composed Score								
2. Montreal Cognitive Assessment	−0.401**							
	−0.438							
3. CES-D Depression Scale	0.317**	−0.152						
	0.248**	−0.201*						
4. Perceived Stress Scale	0.294**	−0.162	0.416**					
	0.238*	−0.187	0.375**					
5. Global Self-Esteem Scale	0.085	−0.085	0.093	0.398**				
	0.036	−0.114	0.060	0.375				
6. General Self-Efficacy Scale	−0.274**	−0.322**	−0.278**	−0.453**	−0.251**			
	−0.161	−0.252*	−0.258**	−0.439**	−0.256**			
7. Attitudes to Aging Questionnaire	−0.332**	0.205*	−0.321**	−0.385**	−0.315**	0.302**		
	−0.221	0.285**	−0.271**	−0.337**	−0.269**	0.254**		
8. Satisfaction With Life Scale	−0.204**	−0.008	−0.315**	−0.307**	−0.238*	0.223*	0.381**	
	−0.212*	−0.266**	−0.330**	−0.294**	−0.212*	0.221*	0.379**	
9. Subjective Face Happiness Scale	−0.182	−0.034	−0.237*	−0.024	0.266**	0.027	−0.180	0.072
	−0.240*	−0.249*	−0.280**	−0.045	0.243*	0.055	−0.144	0.113

Significant at *p ≤ 0.01; *p ≤ 0.05. Partial correlation values are expressed in underline of each variable, controlling for marital status, height, and comorbidity. CES-D, Depression Scale.

Supported by the evidence presented in the correlational analyses, multiple linear regression analyses were used to explore the relationships between the dependent variable of PF composed score and the independent variables of PwB indicators as shown in Table 4. The dimensions of RSES and GSES were not introduced in this analysis as these were not correlated with PF. A hierarchical stepwise model was used, considering the theoretical assumption that cognitive profile and depression state presented a close relationship with frailty (Buchman and Bennett, 2013; Lohman et al., 2016). The results in Table 3 showed that, as expected, the cognitive profile of MoCA explained 22% of the variance by itself (model block 1). Both unadjusted [*F*(6.100) = 11.613; *p* < 0.001; *R*^2^ = 0.340)] and adjusted [*F*(9.97) = 6.789; *p* < 0.001; *R*^2^ = 0.401)] regression analysis (model block 3) models were statistically significant. Observing model (block) 2, the entry of the depression state of CES-D variable did not change the significance values of the MoCA. In model 3, cognitive status (using the MoCA), the HFS score, and the score of the AAQ showed a significant independent relationship with PF in both the unadjusted and the adjusted models (explaining 34 and 40% of variance, respectively). Stress, satisfaction with life, negative mood of depression, and self-efficacy did not significantly contribute to the model. The results indicated that decreased cognition, self-efficacy, and happiness were accompanied by an increased likelihood for being frail. In regression model 3, the strengths of the associations found were attenuated in the adjustment models after the entry of possible bias factors; even so, they were preserved.

**TABLE 4:** The association of psychological well-being indicators and physical frailty composed score (*n* = 358).

**Regression models**	**Physical frailty composed score^a^**					
	**Unadjusted**			**Adjusted**		
	***R*^2^**	***B* coefficient**	***p* value**	***R*^2^**	***B* coefficient**	***p* value**
Model (block) 1	0.22			0.29		
Montreal Cognitive Assessment		−0.467	0.000		−0.452	0.000
Model (block) 2						
Montreal Cognitive Assessment	0.30	−0.440	0.000		−0.420	0.000
CES-D Depression Scale		0.169	0.052	0.32	0.169	0.163
Model (block) 3						
Montreal Cognitive Assessment		−0.378	0.000		−0.369	0.000
CES-D Depression Scale		0.011	0.909		0.028	0.771
Perceived Stress Scale	0.34	0.143	0.157	0.40	0.112	0.264
Satisfaction With Life Scale		−0.024	0.809		−0.024	0.798
Happiness Face Scale		−0.188	0.032		−0.198	0.022
Attitudes to Aging Questionnaire		−0.212	0.034		−0.209	0.038

*Hierarchical stepwise regression model was used and unadjusted bivariate model 1 included PF total score and PwB indicators. Model 2 was further adjusted for variables of height, marital status, and comorbidity. ^*a*^Varies from zero to five points.*

## Discussion

The purpose of this paper was to examine the relationship between indicators of PF and PwB. Firstly, we verified the PF differences in PwB indicators, and the results indicated that frail individuals had a poor satisfaction with life, poor attitudes to aging, poor general self-efficacy, and a heightened state of depression and perceived stress. Based on the relationship of depressive and mood states and cognitive status symptoms, additional PwB variables were investigated to explain the incremental variance in PF scores. Besides the expected effect of the cognitive profile, the results showed that not depressive mood states but a negative attitude to aging and low feelings of happiness proved to independently contribute to the variance in PF status. As far as our knowledge allows, this is the first scientific evidence for the association of PwB health-related domains with PF status in a Portuguese institutionalized female population over 75 years old.

### Comparison by Frailty Subgroups

In agreement with other studies using samples with similar attributes, PF had a similar prevalence (46%) when compared with other European countries who studied population samples living in nursing homes (González-Vaca et al., 2014). The general health was poor and the comorbidities presented with high scores in the frail subgroups, showing that a possible overlap between morbidity and frailty exists (Wong et al., 2010). Interestingly, the sociodemographic of height (but not weight) and marital status (more widowed or divorced) presented with worse results in the frail subgroup. The trend for a reduction in height values in the group of frail elderly could be related to osteopenia/osteoporosis, leading to loss of height (Johansen et al., 2007). This relationship was independent of age in the statistical model and needs to be further explored. Marital status has also been shown in several longitudinal studies to be a powerful predictor of a number of chronic diseases (Lunenfeld and Stratton, 2013) and seems to follow the same trend toward the PF condition.

An analysis of PwB indicators showed that higher scores were found with an increased incidence of PF. This was similar in the Canadian Aging Study (CAS), the results of which revealed that the phenotype of frailty was in an intrinsic relationship with low levels of subjective well-being. The authors of CAS suggested that the low levels of PwB impaired by a frailty syndrome may play an important role in describing the subjective perception of health in older individuals (Andrew et al., 2012). A more recent longitudinal study carried out in a United Kingdom population also found that a higher feeling of PwB was associated with a sense of control, self-realization, and autonomy and may exert a protective effect against PF (Gale et al., 2014). Despite the differences in populations and the different protocols for the evaluation of frailty, these studies were unanimous in confirming the link between frailty status and low general PwB.

Looking at the results of the Bonferroni test after the comparison analysis, it seems that the transition from the non-frailty to the pre-frailty condition is the most critical period for the development of negative outcomes in several PwB dimensions. Some studies indicate that this may also be the most critical stage for the appearance of cognitive decline (Furtado et al., 2018). Apparently, the period of critical intervention for the manifestation of some negative outcomes would be the period attending the transition between frailty and pre-frailty status. The identification of this period would be a primary preventive measure against the arrival of early CF (Ruan et al., 2015), which is currently characterized as one of the outcomes entailing more expenditure for public health.

### Relationships Among PF and PwB

Several PwB indicators were found to be directly associated with the PF composed score. A recent study showed a clear interconnection between PF status and a set of PwB outcomes, highlighting self-efficacy, anxiety, depression, and resilience (Freitag and Schmidt, 2016), but unlike our results, in this study, depression emerged as an important psychological domain that explained the variance in PF scores. In the regression analysis of the present study, a satisfactory relationship explained the PwB variance of PF, and the covariates only had a slight attenuating effect on these relationships. It may be that cognitive status (and an ability to explore and analyze negative feelings) explained the association of depression, self-efficacy, and stress with frailty in our sample. Similar to Campbell and Bucher’s findings 20 years ago, this study and others found that the MoCA independently predicted PF (Lerner et al., 2015). CF is already a widely accepted concept, as is the temporal similarity between the onset of cognitive decline and subsequent deficit in physical function (Kelaiditi et al., 2013). Other factors associated with PF, such as perceived stress and self-efficacy, did not contribute to the regression models in this sample. However, these indicators play an important role in the establishment of the indirect relationships with PF.

The interconnections between stress and physical health remain the most widely studied under a biological approach (Corazza et al., 2013). However, it is possible that several psychosocial events exist, activating emotional stressors with aging. Also, the ability to cognitively adjust to these events and reduce stress and improve self-efficacy to deal with stressors could mediate the relationships found. Attitudes to aging, subjective feelings of happiness, and their association with PF appeared as surprising findings. The attitudes toward aging played an important role in the regression model. A robust cross-sectional survey that collected data in 20 countries and was carried out by a WHO quality-of-life research group showed that attitudes to aging mediated the associations between satisfaction with ones’ health including quality of life, psychosocial, physical, and environmental health (Low et al., 2013). These associations represent robust evidence since the AAQ is a multidimensional construct, which includes three sub-dimensions of psychosocial loss that reflect a high perception of negative feelings; PwB growth is related to the increase of positive feelings regarding life events and physical change, accentuating on items largely associated to health and to the experience of aging itself and consequently resulting in an individualized PwB perception viewpoint that affects physical health (Laidlaw et al., 2007).

In this study, happiness was shown to be an additional factor to explain PF. Positive psychology in recent years has advocated for the assessment of happiness rather than only assessing negative mood and its associations with general health status (Jones et al., 2003). Our data suggest that positive mood may have a more satisfactory contribution to PF rather than a negative mood which may have been explained by other factors present in the model. Interestingly, satisfaction with life and self-esteem were not associated with frailty. Experimental studies including those which can improve mood, such as regular exercise, will show whether our findings may reflect causality. If this is the case, it may be that, through exercise or other activities that improve mood and perceived coping styles (reducing stress and possibly a related increase in self-efficacy and self-esteem), improved attitudes to aging (and possibly the related life satisfaction) will also improve and mediate improvement in PF symptoms.

The take-home significant message of this study is that increasing evidence supports the protective features of the maintenance of a stronger sense of PwB, which may help to reduce the risk for PF and support a reasonable end-of-life-course. Carol Ryff, who has substantial expertise of PwB domains, makes clear the importance of introducing new concepts to help understand the links between the aging process and PwB, highlighting attitude and resilience (Ryff, 2014). Currently, these are key psychological skills for the development of the capacity to maintain or recover good feelings of PwB when facing everyday challenges and difficulties.

### Study Limitations

Despite a construct of satisfactory evidence, this study had some limitations. Firstly, these lie within the sample characteristics, which included more fit individuals than frail people and thus could have caused biased results. Our study is limited to the female sample. In the pilot study, we recorded a small participation of older men. In addition, the percentage of women living in institutions and homes in Portugal is much higher than men, 78 and 22%, respectively. These values made us focus our study on older women. Furthermore, this study has a cross-sectional design and the associations may be bidirectional, and causal reasoning is difficult here as those with PF, because of their limitations, may be more likely to feel less in control, more stressed, and have a more negative attitude and lower feelings of happiness in life. However, the results of the present study showed a similar trend to the other studies with larger samples and those that had a longitudinal follow-up.

### Practical Applications

A meaningful interconnection with important markers of PwB dimensions and PF phenotype was demonstrated in this study. Apparently, PF shows a strong relationship with cognitive aspects, but it also showed a consistent relationship with some emotional dimensions. The transition from frailty to pre-frailty appears to be the most critical period of PwB decline. In this sense, implementing active lifestyle interventions that take into consideration markers of positive feelings in geriatric assessments will assist in the patients physical and mental health care planning as well as prevent the early CF. In this context, it seems that physical–motor activity programs, for example, can help elderly people living in health care and social welfare centers to assist in a possible psychological readjustment in the face of a more secluded lifestyle.

## Conclusion

Overall, the results show that PF was related to poor scores of PwB indicators in institutionalized older women. However, the novelty in this research is the fact that self-perception (attitude toward aging) and emotional well-being (feelings of subjective happiness) were revealed as independent negative predictors of PF since the global cognition performance had already demonstrated strong associations with PF in other studies. It will be necessary in the nearby future to investigate gender differences between these or similar variables and, in addition, to introduce some biological variables in the statistical model in order to test the possible mediators of these relationships.

## Data Availability Statement

The raw data supporting the conclusions of this article will be made available by the authors, without undue reservation.

## Ethics Statement

The studies involving human participants were reviewed and approved by the CE/FCDEF-UC/000202013. The patients/participants provided their written informed consent to participate in this study.

## Author Contributions

GF organizing and drafted the manuscript and worked in the acquisition of data. RL, AV-P, and AC helped in the discussion. EH assisted in the interpretation of data, made additional statistical analysis, and contributed to the critical revision of the content. JF and AT coordinated the research, revised the final version of the manuscript, and added some considerations.

## Conflict of Interest

The authors declare that the research was conducted in the absence of any commercial or financial relationships that could be construed as a potential conflict of interest.
